# ACC deaminase-producing endophytic fungal consortia promotes drought stress tolerance in *M.oleifera* by mitigating ethylene and H_2_O_2_

**DOI:** 10.3389/fpls.2022.967672

**Published:** 2022-12-22

**Authors:** Bushra Rehman, Javeria Javed, Mamoona Rauf, Sumera Afzal Khan, Muhammad Arif, Muhammad Hamayun, Humaira Gul, Sheza Ayaz Khilji, Zahoor Ahmad Sajid, Won-Chan Kim, In-Jung Lee

**Affiliations:** ^1^ Center of Biotechnology and Microbiology, University of Peshawar, Peshawar, Pakistan; ^2^ Department of Botany, Abdul Wali Khan University Mardan, Mardan, Pakistan; ^3^ Department of Biotechnology, Abdul Wali Khan University Mardan, Mardan, Pakistan; ^4^ Department of Botany, Division of Science and Technology, University of Education, Township, Lahore, Pakistan; ^5^ Institute of Botany, University of the Punjab, Lahore, Pakistan; ^6^ Department of Applied Biosciences, Kyungpook National University, Daegu, Republic of Korea

**Keywords:** plant-microbe interaction, *Moringa oleifera*, drought stress, antioxidants, agroforestry, hydrogen peroxide, ethylene, ACC deaminase

## Abstract

**Introduction:**

Drought has become more prevalent due to dramatic climate change worldwide. Consequently, the most compatible fungal communities collaborate to boost plant development and ecophysiological responses under environmental constraints. However, little is known about the specific interactions between non-host plants and endophytic fungal symbionts that produce growth-promoting and stress-alleviating hormones during water deficits.

**Methods:**

The current research was rationalized and aimed at exploring the influence of the newly isolated, drought-resistant, ACC deaminase enzyme-producing endophytic fungi *Trichoderma gamsii* (TP), *Fusarium proliferatum* (TR), and its consortium (TP+TR) from a xerophytic plant *Carthamus oxycantha* L. on *Moringa oleifera* L. grown under water deficit induced by PEG-8000 (8% osmoticum solution).

**Results:**

The current findings revealed that the co-inoculation promoted a significant enhancement in growth traits such as dry weight (217%), fresh weight (123%), root length (65%), shoot length (53%), carotenoids (87%), and chlorophyll content (76%) in comparison to control plants under water deficit. Total soluble sugars (0.56%), proteins (132%), lipids (43%), flavonoids (52%), phenols (34%), proline (55%), GA_3_ (86%), IAA (35%), AsA (170%), SA (87%), were also induced, while H_2_O_2_ (-45%), ABA (-60%) and ACC level (-77%) was decreased by co-inoculation of TP and TR in *M. oleifera* plants, compared with the non-inoculated plants under water deficit. The co-inoculum (TP+TR) also induced the antioxidant potential and enzyme activities POX (325%), CAT activity (166%), and AsA (21%), along with a lesser decrease (-2%) in water potential in *M. oleifera* plants with co-inoculation under water deficit compared with non-inoculated control. The molecular analysis for gene expression unraveled the reduced expression of ethylene biosynthesis and signaling-related genes up to an optimal level, with an induction of antioxidant enzymatic genes by endophytic co-inoculation in *M. oleifera* plants under water deficit, suggesting their role in drought stress tolerance as an essential regulatory function.

**Conclusion:**

The finding may alert scientists to consider the impacts of optimal reduction of ethylene and induction of antioxidant potential on drought stress tolerance in *M. oleifera.* Hence, the present study supports the use of compatible endophytic fungi to build a bipartite mutualistic symbiosis in *M. oleifera* non-host plants to mitigate the negative impacts of water scarcity in arid regions throughout the world.

## Introduction

Plants being sessile are vulnerable to a variety of environmental stresses, including low temperature, excessive salt, and drought, which progressively limit plant growth and development (Dawood et al., 2022). From seed germination to senescence, ethylene is one of the fundamental phytohormones and regulators of plant developmental and physiological processes. It also serves as an important signaling molecule in the abiotic stress tolerance mechanism whereby plants control ethylene to activate signaling pathways that protect them from the negative effects of abiotic stress (Sadiq et al., 2020). Many studies have found that ethylene or its precursor 1-aminocyclopropane-1-carboxylate (ACC) improves stress tolerance in a variety of plant species, including corn, Arabidopsis (Rauf et al., 2013), tomato (Gharbi et al., 2017), grapevines (Sun et al., 2016), and wheat (Rauf et al., 2021). Other studies, however, stated that ethylene played a detrimental influence on the growth and development of plants including tomato, Cucurbita pepo, tobacco, and Arabidopsis, under abiotic stress (Cebrián et al., 2021). Interestingly, this differential impact of ethylene in response regulation to abiotic stress is dependent on its optimal biosynthesis level in plant tissue and plant susceptibility to it, as the ideal ethylene level for proper plant development varies at different stages and in different plant species (Tao et al., 2015).

Ethylene biosynthesis initiates by precursors S-adenosyl-L-methionine (SAM) and 1-aminocyclopropane-1-carboxylic acid (ACC), and the key synthesizing enzymes catalyzing this route including SAM, ACC synthase (ACS), and ACC oxidase (ACO) (Kende, 1993). Ethylene production in response to a variety of environmental constraints is suggestive of a link between environmental change and developmental adaptability. Moreover, ethylene as a signaling molecule influences the chemical linkages between other phytohormones and plant growth. Flooding, submergence, shade, heat, heavy metal exposure, salt, reduction in nutrient availability, and drought are paradigms of abiotic constraints that induce ethylene production (Rauf et al., 2013a; Dubois et al., 2017; Savada et al., 2017).

Drought is a severe threat and a highly unanticipated constraint, hindering crop cultivation and agroforestry worldwide. It is inevitable to avoid the water deficit for plants growing in arid zones, as drought is limiting plant development and agricultural production by interfering with plant growth from germination through maturity (Han et al., 2020). Some plants have acquired defense mechanisms (morphological, physiological, and molecular flexibility and adaptation) against stressors during the evolutionary process. Such drought-tolerant plants are known as xerophytics due to the adaptive mechanisms to endure water deficit, such as biogenesis and accumulation of osmolytes in controlling the turgor pressure, which averts structural membrane injury, regularizes ionic homeostasis, modulates water uptake and use efficiency, flexibility in overall growth and its plasticity (morpho-physiological flexibility and plasticity, enhanced photosynthetic and antioxidant potential, phytohormones biosynthesis and reshuffling, as well as inherited adaptability to modify the gene expression that controls stress signaling upon perception to provoke the metabolite and hormonal biosynthesis and signaling pathways for ultimate stress alleviation process (Bernstein, 2019). Nonetheless, these modifications are insufficient to provide endurance against globally prevalent drought, since not all plant species are fully capable of dealing with water deficit (Kogan et al., 2019). In addition to this, not all plant species can restore their natural and optimal hormonal level required for survival and growth under stressful environments. As a result, water deficit has been demonstrated to have a detrimental impact on the growth and development of plants (Sadak et al., 2020).

Researchers have been investigating several ways that may be effective in improving drought tolerance in plants. Apart from investigating the morpho-physiological changes, multiple gene expression studies have been useful to understand drought-responsive processes in various crops. Genetic engineering looks to be a powerful technique for developing ideal plants with desired characteristics. Investigating the molecular drought stress tolerance-associated pathways may thus be beneficial for enhancing stress resistance efficiency in plants. Recent developments in high-throughput sequencing technology have expedited genetic research by lowering the cost of genome-wide marker discovery dramatically. This allowed a genome-wide association study (GWAS) to be used to analyses the genetic mechanism underlying agronomically significant characteristics and find marker-trait associations (MTAs) to be employed in breeding programmed to generate superior cultivars with biotic and abiotic stress-resistant traits. GWAS has been conducted in maize, sorghum, rice, perennial ryegrass, and tall fescue, for the selection and improvement of desirable phenotypic traits (Talukder et al., 2021).

Moreover, higher quality of the genome assemblies of significant plants is need of the time for extracting the valuable genomic traits and stress-resistant marker genes for crop development through genetic engineering. In addition, genomes and transcriptomes have provided vital resources to aid the growth and development of plants under stress. *M. oleifera* is a well-known plant species for its nutritional and immunity-boosting properties. Shyamli et al. (2021) have recently performed genome assembly of *M. oleifera* var. Bhagya by long reads (PacBio) and short reads (Illumina) to generate better genome coverage. The phylogenetic study indicated that *M. oleifera* has drought-induced ethylene biosynthesis and signaling genes related to closest orthologue genes from *A. thaliana* (*DREB1A, ETO1 RAP27, ACS8), O. sativa (CRL5)*, and *P. mume (ACCO).* Moreover, orthologue genes for antioxidant enzymes from *A. thaliana* were also found in *M. oleifera*, such as *PER43, CATA2*, and *GPX2.* In many plants, the conserved nature of these stress-resistant marker genes with their preserved biochemical role in the drought tolerance responses throughout evolution may recognize them as a crucial part of *M. oleifera* as well, to be exploited for drought stress tolerance induction.

Another method used by researchers to trigger drought resistance in *M. oleifera* is the use of external priming facilitators (Ezzo et al., 2018). Though, the application and manufacture of these substances are time-consuming, expensive, and, in most cases, hazardous to the natural environment. Furthermore, the efficacy of synthetic fertilizers, chemical-based priming facilitators, and growth inducers differs depending on environmental constraints and plant nature.

The use of extreme-habitat-adapted symbiotic endophytic fungi is another well-known strategy for minimizing the detrimental effects of abiotic stresses on crops. Drought and other harsh environmental conditions have increased the chance of exploring the fungal endophytes capable of inculcating drought resistance in non-host plants. Therefore, scientists have adapted this promising substitute method of utilizing an eco-friendly, low-cost, and non-toxic organic source such as endophytic fungi for persuading drought-stress tolerance as well as effective growth promotion through modulation of phytohormonal biosynthesis, antioxidant potential, and water-retaining capability of plants.

Keeping in view the previous knowledge about the drought stress responses in the role of endophytic fungi, the present research findings enabled us to decipher the rebalancing of the biochemical, physiological, metabolic, antioxidant, phytohormonal, and molecular mechanisms by the influence of endophytic fungal interaction in *M. oleifera* for alleviation of drought stress. The main rationale of the current research was to unravel the novel drought stress-resistant endophytic fungi from a xerophytic host and investigate the effect of newly isolated ACC deaminase-producing endophytic strains TP and TR, on *M. oleifera* plants under PEG-induced drought stress.

## Materials and methods

### Identification, purification, and characterization of endophytic fungi from the host plant

Endophytic fungal isolation and purification were done from the host plant *Carthamus oxyacantha* L. obtained appropriately from the arid region of Shenki Jalala area, Takht Bhai (34°20’8 N 71°54’10 E) at a height of 339 m (1115 ft), Khyber Pakhtunkhwa Pakistan.

Five fungal strains from stem tissue were isolated and purified on the PDA medium as described previously (Javed et al., 2022) and subsequently stored in the refrigerator till further characterization and exploitation.

### Macro and microscopic phenotypying of fungal strains

The apparent morphological characteristics of fungal strains were used to assess them initially. Erdman’s (1952) standard protocols were used for fungal strain microscopy. A small part of the fungal specimen was isolated and placed on the glass slide, shielded with a coverslip to flatten the sample, and visualized at the magnification, i.e., 40x and 100x under the light microscope (Binocular NSL - CX23 Olympus, Japan). Lactophenol cotton blue reagent was utilized to stain and mount the medium for microscopic examination of the fungal parts as described by Shamly et al. (2014).

### Assessment of drought resistance trait of fungal endophytes

All pure strains (five) from stem tissues were chosen for preliminary screening, following the water deficit generated by supplementing PEG-8000 (8%), as osmoticum to imitate the osmotic pressure of fungal cells using 50 mL of czapek medium. Among all endophytic fungi, the two strains designated TP and TR were chosen as water deficit-resistant in an 8% PEG-supplemented medium. The endophytic fungus secretes a variety of metabolites to collaborate with the host plant species to promote growth. Plant hormones (IAA, GA, ABA, ACC; ethylene precursor, and SA), primary/secondary metabolites, and antioxidants (enzymatic and non-enzymatic) were all evaluated in fungal culture filtrate. For plant inoculum preparation, the final concentration of spore suspension (∼5x10^7^ spores/mL) was maintained. The supernatant and biomass were separated using sterilized Whatman filter paper followed by centrifugation (Sartorius Modle: 2-16 PK) at 4000 × g, 4°C, for 15 min. The supernatants were then frozen and stored at -80°C refrigerator for further procedures.

### Strain identification

The chosen endophytic fungal strains (TP and TR) were identified at the molecular level by amplification of ITS region of *18S rRNA* gene with primer pair (F-ITS-1/R-ITS-4) (Lee Taylor and Bruns, 1999). A total of 30 μl of PCR reaction was at least 20 ng of genomic DNA as a template. The cloned and purified PCR products were sequenced by BGI Co. Ltd (Shenzhen, China) using universal primers, as mentioned earlier (Rauf et al., 2021). The reverse and forward reads of sequenced fragments were aligned using a coding codon aligner (version 7.2.1, Codon code corporations), and the resultant homolog sequence was submitted to nucleotide BLAST query in the NCBI (http://www.ncbi.nlm.nih.gov/BLAST) database. The nearest homolog sequences were extracted and evaluated using MEGA 7 (version 7.0.18), for phylogenetic analysis. Sequences (ITS region) of identified isolates TP and TR were submitted to NCBI GenBank.

### ACC deaminase gene and enzyme activity of TP and TR endophytic fungal strains

ACC deaminase activity was determined according to the method of Yedidia et al. (1999) as described by Zhang et al., 2019. To this end, fresh fungal culture filtrate was used, and ACC deaminase activity was quantified spectrophotometrically by measuring the absorbance of the final product (ketobutyrate) at 540 nm.. For that purpose, 1 ml of TP and TR spore solution (1x10^8^ spores’ ml ^1^) was added to the synthetic medium (50 mL), with the addition of the ACC (0.5, 1.0, 1.5, and 2.0 mM).

*ACCD* gene expression was evaluated by Real Time-quantitative PCR (RT-qPCR) as described earlier by Viterbo et al. (2010). Total RNA was extracted from each fungal sample and DNAase-treated RNA samples were purified with RNeasy Mini columns (Qiagen, Hilden, Germany). An equal amount of RNA (2 µg) was used to prepare the first strand cDNA using SuperScript II reverse transcriptase (Invitrogen, Lyon, France) and oligo (dT) as a primer, as mentioned by the manufacturer’s protocol. SYBR Green (Applied Biosystems Applera, Darmstadt, Germany) to perform RT-qPCR using ABI PRISM 7900HT (Applied Biosystems Applera, Darmstadt, Germany).

### Analysis of the TP and TR endophytic fungal culture filtrate

Seven days old fungal culture filtrate was used for biochemical, hormonal, and metabolic quantifications. Total phenols and flavonoids were measured in culture filtrate as described earlier (Khatiwora et al., 2017). The culture supernatant (0.2 ml) was added with Folin-Ciocalteu reagent (0.8 ml) (Sigma Aldrich, Burlington, MA, USA) and Na_2_CO_3 (_2 _ml_ of 7.5%) (Sigma Aldrich, Burlington, MA, USA), with subsequent dilution of the samples by adding 7 volumes of dH_2_O and incubation was done in dark for 2 hours. Catechol (1 to 10 mg) (CellMark AB, Göteborg, Sweden) was used to generate a standard curve, and absorbance was measured at 650 nm with a spectrophotometer (UV/VIZ spectrophotometer; PerkinElmer Inc., USA). Total proteins (Lowry et al., 1951), total soluble sugar content (Mohammadkhani and Heidari, 2008), lipids (Vogel, 1956), and proline (Bates et al., 1973) were all determined. The Salkowski reagent was used to calculate the amount of indole-3-acetic acid (IAA) in the fungal filtrate (Benizri et al., 1998). Gibberellic acid, Abscisic acid (Ergün and Topcuoğlu, 2002), Salicylic acid (Warrier et al., 2013), and Ascorbic acid (Asada, 1992) were quantified as described earlier. In the culture filtrate, oxidative enzymes such as Catalase (Chandlee and Scandalios, 1984), total antioxidants (Yen and Chen, 1995), peroxidase activity (Malik, 1980), and H_2_O_2_ (Moloi and van der Westhuizen, 2006) were measured.

### Application of TP and TR endophytic fungi on *M. oleifera* under drought stress

*M. oleifera* seeds were collected from National Agricultural Research Centre (NARC), Islamabad (33°41′35″N/73°03′50″E), Pakistan. The surface sterilized, stratified seeds were placed on filter papers, imbibed, and kept for 6 days at 4°C. Drought stress induction was done using PEG-8000 (8%). While *TP* and *TR* endophytic fungal application were done by single or combined inoculation of an equal volume of culture filtrate to imbibe the seeds.

To assess the response of *M. oleifera* to PEG-8000-induced osmotic/water deficit in a natural environment, 3 days old, uniformly germination seedlings were transferred to the autoclaved soil pots (1 seed/pot), pre-mixed with TP and TR fungal biomass (3 g/pot). Control pots, on the other hand, lacked active culture biomass and culture filtrate.

The endophytic fungus spore suspension was injected at 1 mL/seedling of each pot 14 days after germination. The density of the spore solution was increased to 5x10^7^ spores/mL. For water deficit induction, 3 ml of PEG-8000 (8%) was administered to the base of evenly growing *M. oleifera* seedlings on alternate days for 15 days following germination.

The 21-day-old seedlings were subjected to a drought induction by supplementing 8% PEG-8000 with 3 mL per pot for 6 days, followed by a recovery period of 9 days. The experimental setup included three biological replicates, each with 16 pots (6 cm diameter/8 cm height) with consistently one plantlet per pot. For each treatment, pots were filled with 400 g of sterilized sand/soil (8:2 proportion). Various treatments used in this research have been mentioned in **Supplementary Table 1**. The plants were grown in the natural environment from March 2021 to May 2021 (20 ± 2.4°C to 37 ± 3.2°C) in the Abdul Wali Khan University Mardan Botanical Garden (34° 11’ 54” North, 72° 2’ 45” East).

### Analysis of growth attribute in *M. oleifera* under drought stress

For the assessment of growth response, *M. oleifera* L. growth parameters such as seed fresh weight of seedlings, total cotyledonary length, total shoot, and root length, and fresh and dry weight, were measured.

### 3, 3-diaminobenzidine assay

Protocol from Thordal-Christensen et al. (1997) was used for the 3, 3-diaminobenzidine (DAB) test to visualize the H_2_O_2_ accumulation. Leaf segments of 1 cm length from the 4^th^ compound leaf of 35-day-old seedlings were vacuum-filtered with DAB staining solution. To eliminate chlorophyll content, samples were incubated in 90% ethanol for 10 minutes at 70°C. To avoid auto-oxidation, the DAB working solution was newly made (Fryer et al., 2002). Similarly, DAB polymerization caused H_2_O_2_ to appear brown, and finally, leaf segments were visualized at the magnification, i.e., 40x under the light microscope (Binocular NSL - CX23 Olympus, Japan).

### Biochemical analysis in *M. oleifera* under drought stress

The total chlorophyll contents of *M. oleifera* seedlings were determined using the methodology used by Maclachlan and Zalik (1963). 3 mL of 80% acetone was used to grind leaf samples. The total soluble sugar concentrations of *M. oleifera* seedlings were measured using 0.5 g of fresh plant tissue (Mohammadkhani and Heidari, 2008). As described by Lowry et al. (1951), 1 g tissue of fresh leaf samples was taken to assess the protein levels. Total lipids were determined in 1 g of fresh leaf tissue using the procedure (Vogel, 1956).

Using 2 g of fresh leaf tissue, Bates et al. (1973) determined the proline level of *M. oleifera.* The approach was utilized by Khatiwora et al. (2017) to determine flavonoids and phenolic contents in *M. oleifera* using 1 g of fresh leaf tissue.

### Quantification of phytohormones and antioxidant enzyme activities in *M. oleifera*

The level of indole-3-acetic acid (IAA) was assessed by adapting the protocol mentioned by Benizri et al. (1998). The method of (Ergün and Topcuoğlu, 2002) was used to determine abscisic acid (ABA) and gibberellic acid (GA_3_). The Paul and Vinitha (2013) procedure was used to determine the salicylic acid (SA) concentration. The 1-Aminocyclopropane-1-carboxylic acid (ACC) content was measured using the method of Yu et al. (2016).

To quantify catalase activity (CAT), the method proposed by Chandlee and Scandalios (1984) was followed. Ascorbate peroxidase (APX) activity was measured using the approach developed by Asada, (1987).

### RT-qPCR analysis for gene expression in *M. oleifera*

The RT-qPCR analysis was carried out as previously reported (Rauf et al., 2021). The GeneJET Plant RNA Purification Kit (Thermo ScientificTM) was used to extract total RNA from 35-day-old plants (leaf and root tissue). Approximately 2 g of total DNAase-treated RNA was used for reverse transcription using the RevertAid First Strand cDNA Synthesis Kit (Invitrogen, Karlsruhe, Germany). The expression of selected ethylene biosynthesis and signaling genes, as well as antioxidant enzymatic genes, was normalized with the housekeeping gene translation elongation factor 2 (*EF2*) since Deng et al. (2016) cited *EF2* gene for the steady expression under PEG-induced drought stress in *M. oleifera.*

### Statistical analysis

The experiment was carried out using a complete randomized design (CRD). GraphPad Prism 9.0.0 (121) software was used to statistically analyze the data, which represented the means and standard errors of three independent replicates for each treatment, using RM Two-Way Analysis of Variance (ANOVA). The statistical data were double-checked using the statistical software tool SPSS V. 21.0. (SPSS, Chicago IL, USA). Similarly, Duncan’s Multiple Range Test was used to separate the means (DMRT). At p ≤ 0.05, significant differences were depicted by various statistical bars labeled with significant letters.

The principal component analysis (PCA) was accomplished by using OriginPro8 software, to analyze the multivariate effect of drought-tolerant endophytic fungi (TP and TR), and PEG-induced drought stress on plant traits (primary, secondary metabolites, enzymatic and non-enzymatic antioxidants, H_2_O_2_, fresh/dry weight, shoot/root length, water potential).

## Results

### Endophytic fungal isolation and screening for drought tolerance

A total of five strains of endophytic fungi were isolated from the stem segments of the xerophytic plant *Carthamus oxyacantha* L. The strains were grown on czapek medium and screened against PEG-8000 (8%) for drought stress tolerance, as previously described by Javed et al. (2022) and pure cultures were allowed to grow at 30°C, 7 days, 120 rpm shaking incubator. Finally, the growth of isolates was assessed by harvesting the fungal biomass through filtration. The PEG-mediated drought tolerant strains were then tested for various growth parameters before being administered to *M. oleifera* plants subjected to PEG-mediated drought stress. Endophytic fungi TP and TR demonstrated the highest drought tolerance response, by producing the maximum biomass in both the control and stressed conditions and were selected for further experimental procedures and plant bioassays.

**Figure 1:**
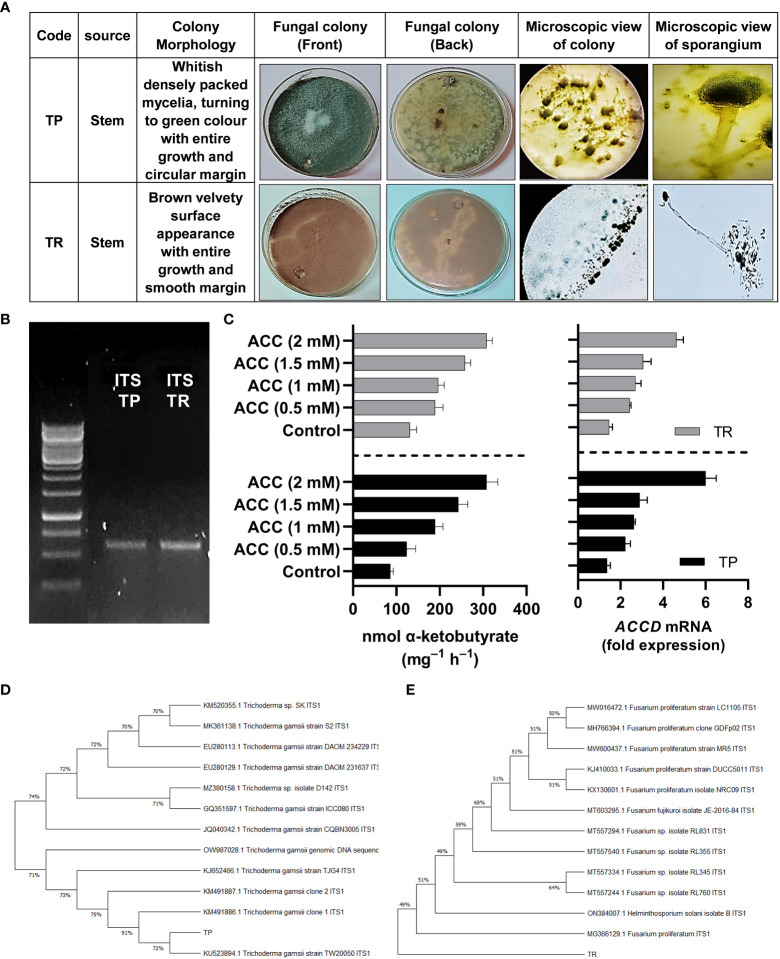
**(A)** Morphological characterization of selected fungal strains TP and TR, **(B)** genotyping of selected fungal strains by ITS region amplification, **(C)** ACC deaminase activity and *ACC deaminase* gene expression of TP and TR. Quantitative data represent means ± *SD* of three independent experiments and at least six technical replicates, **(D)** Phylogenetic identification of TP, and **(E)** TR endophytic strain.

Preliminary identification of selected strains (TP and TR) of fungal endophytes was done based on visual traits such as colony texture, form, color and growth pattern, hyphae color, and spores’ morphology (**Figure 1A**).

### ACC deaminase activity and RT-qPCR for *ACCD* transcript abundance

By growing in ACC enriched Czapek medium, TP and TR exhibited growth and the potential for ACC deaminase activity (ACCD). The ACC deaminase activity of the TP strain was shown to be up to 135-nmol of ketobutyrate mg^-1^ h^-1^, which was later raised to 342-nmol of ketobutyrate mg^-1^ h^-1^. While the ACC deaminase activity of the TR strain was shown to be up to 126-nmol of ketobutyrate mg^-1^ h^-1^, which was later raised to 302-nmol of ketobutyrate mg^-1^ h^-1^ (**Figure 1**). Moreover, ACC deaminase orthologue gene identification in TP and TR isolates was carried out using NCBI gene search browser and RT-qPCR analysis was used to assess transcript abundance, and the results demonstrated that *ACCD* transcript was upregulated in an ACC-dependent manner. The abundance of *ACCD* transcripts was found to be dose-dependent, as illustrated in **Figure 1**. The *ACCD* gene expression was induced up to 36% for the TP strain, and 175% for the TR strain compared with the control (**Figure 1**).

### Molecular identification and phylogenetic analyses based on ITS sequences

For TP and TR isolates, the ITS region of *18 S rRNA* gene was amplified and sequenced (**Figure 1**). After sequencing, the discovered sequence was compared to data in the GenBank sequence database to establish the genus or species of the TP and TR isolates. The sequences used in this investigation may be found in the online repository. After a homology search on GenBank indicated 100% similarity to *Trichoderma gamsii*, the ITS sequence of TP was classified to the species level (TP). As a result, the strain was identified and given the name *Trichoderma gamsii.* TR’s ITS sequence showed 100% similarity to *Fusarium proliferatum*, identifying the TR strain as *Fusarium proliferatum* (**Figure 1**).

The sequences were deposited in NCBI GenBank as accession No. OP419488 for TP and OP419489 for TR isolate.

### Effect of PEG-induced drought stress on the biochemical, hormonal, and antioxidant traits of TP and TR endophytic fungi

**Figure 2:**
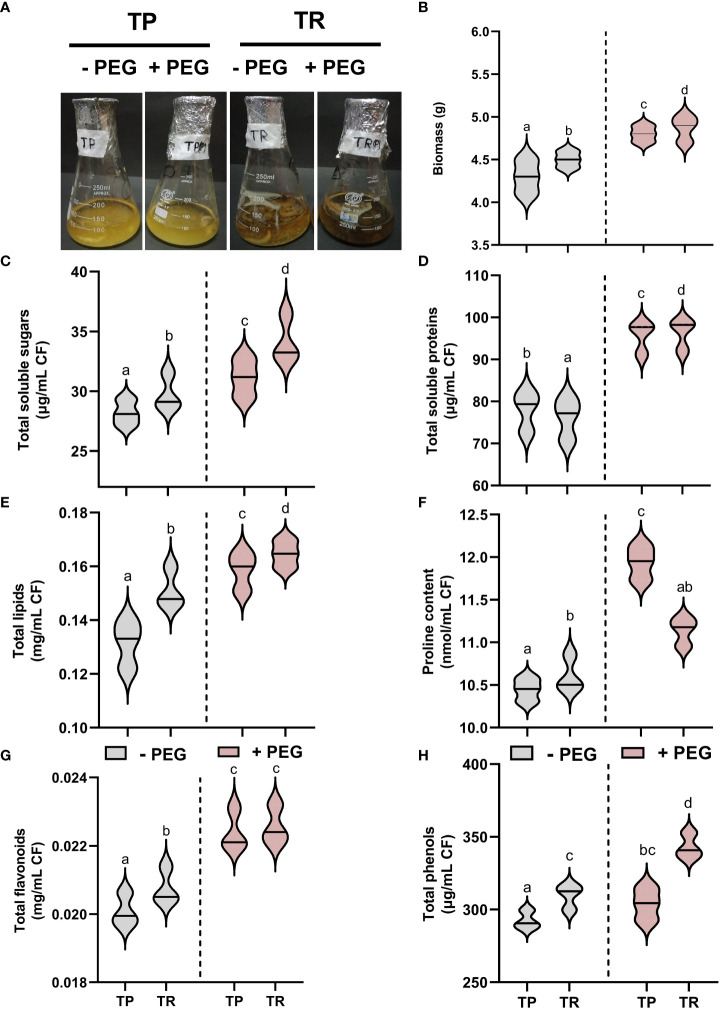
Endophytic fungal (TP and TR) characterization. **(A)** Screening of fungal strains (TP and TR) for PEG-induced drought stress tolerance, **(B)** Biomass, **(C)** Total soluble sugars, **(D)** Total soluble proteins, **(E)** Total lipids, **(F)** Proline content, **(G)** Total flavonoids, and **(H)** Total phenols, measured in CF of TP and TR isolates. Quantitative data is representing the means ± *SE* values of at least three independent biological replications. Various letters have been presented to show the statistical differences at significance level of *p¾*0.05 using Duncan’s Multiple Range Test (DMRT). CF, Culture Filtrate.

Current results showed that endophytic fungal isolate TP significantly (*p ≤* 0.05) overproduced the biomass and generated sufficient content of total soluble proteins (25%), total soluble sugars (12%), lipid content (18%), total phenolics (8%), total flavonoids (121%), and proline content (7%), upon PEG-induced drought stress in comparison to control cultures. Under PEG-induced drought stress, TR isolate also significantly (*p ≤* 0.05) overproduced the total soluble proteins (6%), total soluble sugars (14%), lipid content (14%), total phenolics (13%), total flavonoids (11%), and proline content (11%) than control (**Figure 2**).

**Figure 3:**
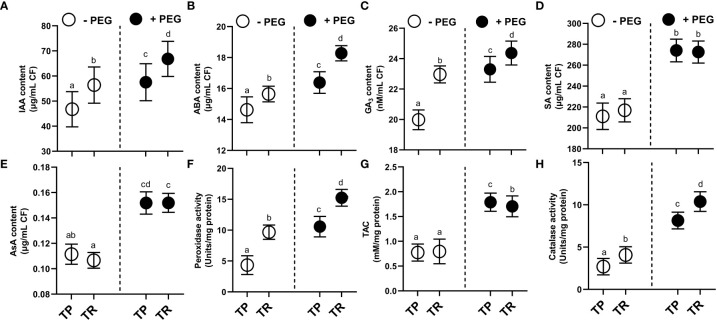
Hormonal contents and enzymatic and non-enzymatic antioxidants in CF of TP and TR isolates. **(A)** IAA, **(B)** ABA, **(C)** GA_3_, and **(D)** SA content. **(E)** AsA content, **(F)** Peroxidase enzyme activity, **(G)** total antioxidant capacity, and **(H)** Catalase enzyme activity. Quantitative data is representing the means ± *SE* values of at least three independent biological replications. Various letters have been presented to show the statistical differences at significance level of *p¾*0.05 using Duncan’s Multiple Range Test (DMRT). CF, Culture Filtrate.

Under both normal and PEG-induced water deficit conditions, TP and TR isolates were shown to generate indole acetic acid (IAA), abscisic acid (ABA), gibberellic acid (GA), and salicylic acid (SA) (**Figures 3A–D**). PEG supplementation significantly (*p ≤* 0.05) raised the level of secreted IAA in culture filtrate for TP (26%) and TR (22%) compared to control. In comparison to the control, TP (8%) and TR (12%) significantly (*p ≤* 0.05) increased the level of secreted abscisic acid (ABA) production in culture filtrate grown under PEG-mediated drought stress. Endophytic fungi TP (15%) and TR (9%) significantly (*p ≤* 0.05) increased the level of secreted GA_3_ production in culture filtrate grown in PEG supplementation, in comparison to the control. Moreover, free SA levels in PEG-supplemented cultures were also significantly (*p ≤* 0.05) increased for TP (21%) and TR (5%) compared to the respective controls (**Figure 3**).

Under PEG-induced water deficit, TP and TR isolate produced antioxidant enzymes with elevated antioxidant capacity (**Figures 3E–H**). In comparison to the control, TP (5%) and TR (6%) significantly (*p ≤* 0.05) increased the content of ascorbic acid (non-enzymatic antioxidant) in culture filtrate under drought stress. PEG supplementation significantly (*p ≤* 0.05) induced the ascorbate peroxidase (POX) activity by TP (100%) and TR (85%), catalase (CAT) activity by TP (129%) and TR (203%), and total antioxidant capacity (TAC) by TP (0.74%) and TR (0.36%) compared with the control culture (**Figure 3**).

### Effect of TP and TR endophytes on growth attributes and growth-related metabolites of *M. oleifera* under PEG-induced drought stress

**Figure 4:**
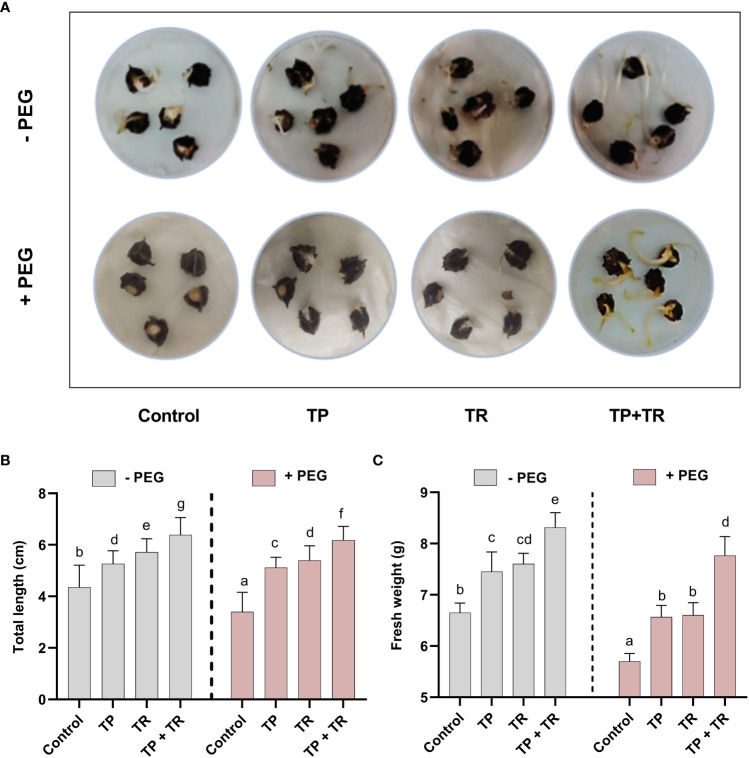
Assessment of PEG-mediated drought stress tolerance response of *M. oleifera* seeds by germination test. **(A)** Seed germination response under PEG-induced drought conditions in the absence (above penal) and presence (lower penal) of endophytic CF inoculation, **(B)** cotyledon length and **(C)** fresh weight. Quantitative data is representing the means ± *SE* values of at least three independent biological replications. Various letters have been presented to show the statistical differences at significance level of *p¾*0.05 using Duncan’s Multiple Range Test (DMRT).

**Figure 5:**
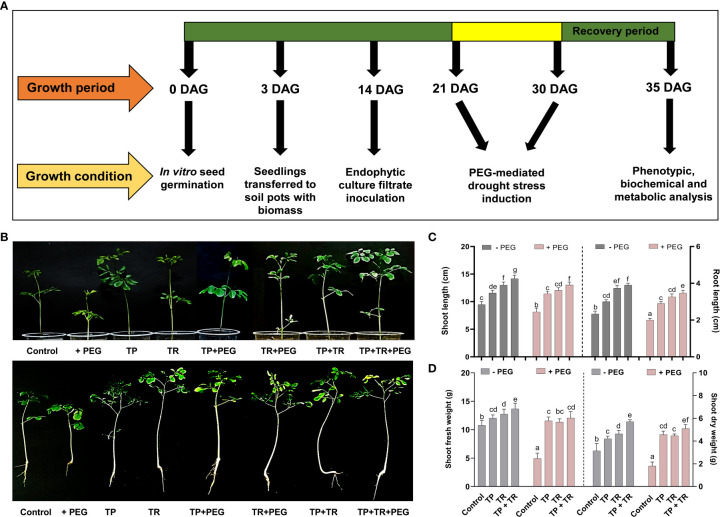
Effect of TP and TR inoculation on *M. oleifera* plant growth. **(A)** Schematic representation showing work plan of plant bioassay, **(B)** Phenotypic analysis; upper penal (intact plants) and lower penal (uproots plants), **(C)** Shoot and root length, **(D)** Total fresh and dry weight. Quantitative data is representing the means ± *SE* values of at least three independent biological replications. Various letters have been presented to show the statistical differences at significance level of *p¾*0.05 using Duncan’s Multiple Range Test (DMRT).

Under field circumstances, the plant growth-promoting potential of fungal inoculations was investigated, in terms of increased fresh weight and cotyledon length measured at 7 days after germination (DAG), as well as shoot and root length, and fresh and dry weight assessed at 35 days after germination, *M. oleifera* plants. Individually, both TP and TR fungal isolates (with and without PEG-induced drought stress), considerably improved growth characteristics. However, combined inoculation of TP and TR isolates, significantly (*p ≤* 0.05) increased the cotyledon length (102%), cotyledon fresh weight (32%), dry weight (217%), fresh weight (123%), root length (65%), shoot length (53%), in *M. oleifera* plants subjected to PEG-induced drought stress vs non-inoculated control plants under stress (**Figures 4**, **5**).

**Figure 6:**
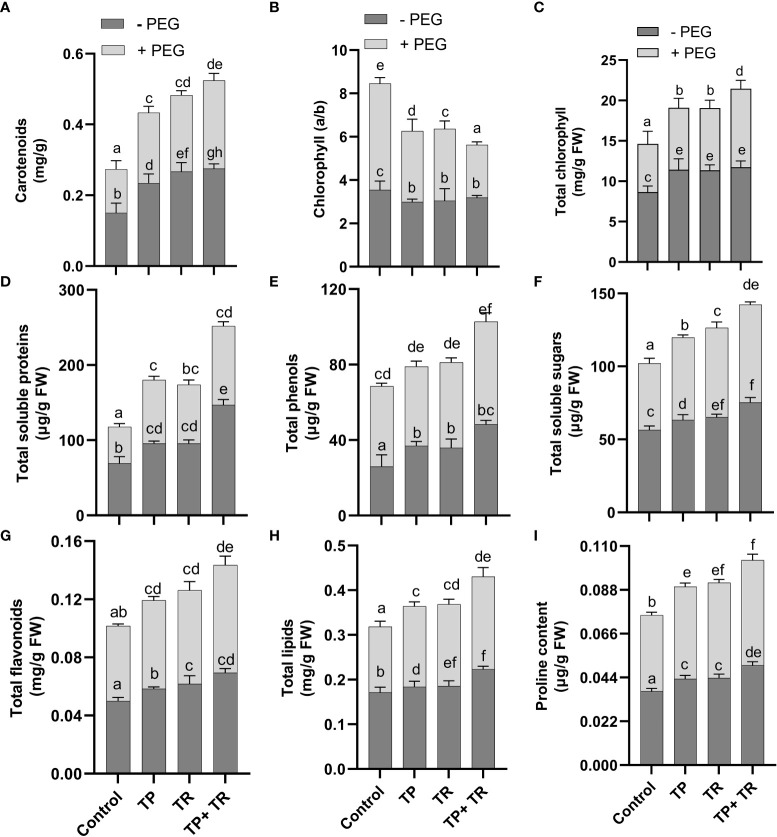
Effect of TP and TR inoculation on *M. oleifera* growth promoting metabolites. **(A)** Carotenoids, **(B)** Chlorophyll a/b ratio, **(C)** Total chlorophyll content, **(D)** Total soluble proteins, **(E)** Total phenols, **(F)** Total soluble sugars, **(G)** Total flavonoids, **(H)** Total lipids, and **(I)** Proline content. Quantitative data is representing the means ± *SE* values of at least three independent biological replications. Various letters have been presented to show the statistical differences at significance level of *p¾*0.05 using Duncan’s Multiple Range Test (DMRT).

Under field conditions, with and without PEG-induced drought stress exposure, both TP and TR individual inoculation significantly (*p ≤* 0.05) increased photosynthetic and accessory pigments (chlorophyll and carotenoid), total soluble protein, total phenolics, total soluble sugars, total flavonoids, lipid content, and proline content in *M. oleifera* plants at 35 DAG, compared to non-inoculated control plants under stress (**Figure 6**). However, TP and TR co-inoculation significantly (*p ≤* 0.05) has enhanced the production of carotenoids (87%), and total chlorophyll content (76%) in *M. oleifera* plants under stress, in comparison to non-inoculated control plants. Contrary to this, both TP and TR individual inoculation significantly (*p ≤* 0.05) has reduced the chlorophyll a/b ratio in *M. oleifera* plants at 35 DAG, compared to non-inoculated control plants under stress (**Figure 6**). Increased values of chlorophyll a/b ratio corresponded to a decline in photosynthetic activity and initial stress symptoms in plants.

Moreover, TP and TR co-inoculation also significantly (*p ≤* 0.05) promoted the production of total soluble sugars (56%), total soluble proteins (132%), lipids (43%), total flavonoids (52%), total phenolics (34%), proline content (55%), in *M. oleifera* plants at 35 DAG, compared to non-inoculated control plants under stress (**Figure 6**).

### Effect of TP and TR endophytes on hormonal contents of *M. oleifera* under PEG-induced drought stress

Under both drought stress and normal condition, endogenous IAA, GA, SA, and ASA levels were significantly (*p ≤* 0.05) raised in response to the individual as well as co-inoculation of TP and TR isolates compared to non-inoculated control plants.

**Figure 7:**
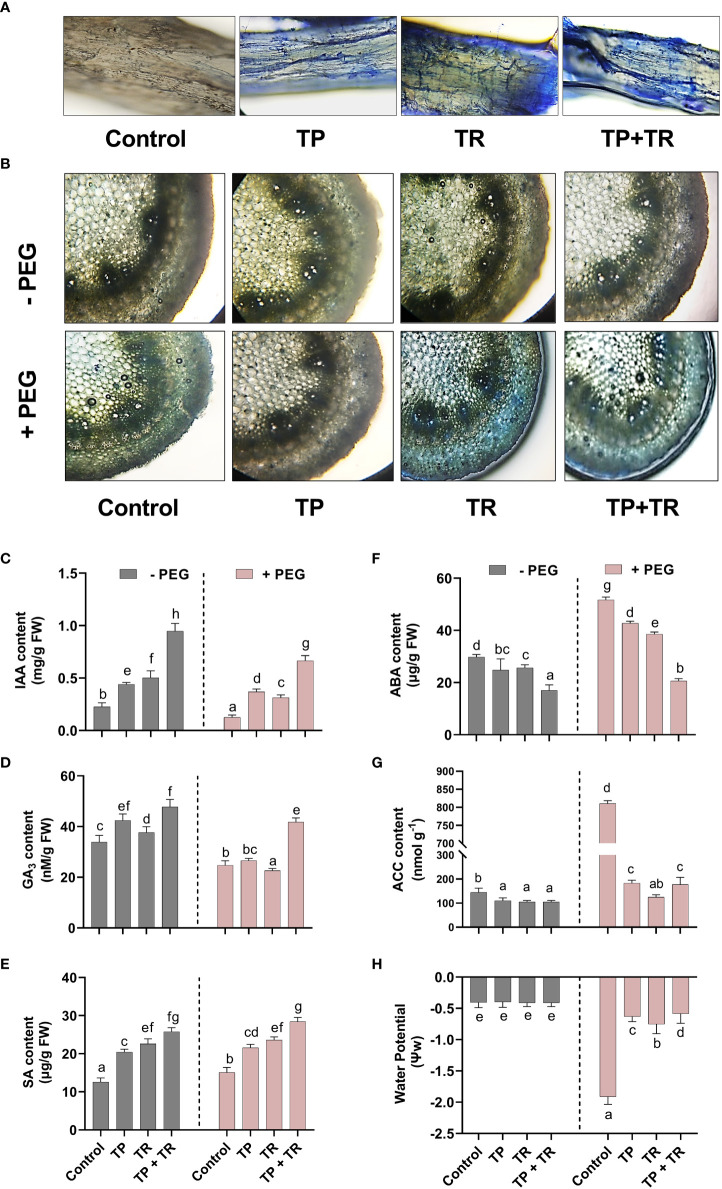
Effect of TP and TR inoculation and colonization in *M. oleifera* phytohormones and water potential. **(A)** Root colonization, **(B)** Stem anatomy, **(C)** IAA, **(D)** GA_3_, **(E)** SA, **(F)** ABA, **(G)** ACC content and, **(H)** Water potential. Quantitative data is representing the means ± *SE* values of at least three independent biological replications. Various letters have been presented to show the statistical differences at significance level of *p¾*0.05 using Duncan’s Multiple Range Test (DMRT).

TP and TR co-inoculation significantly (*p ≤* 0.05) enhanced the production of GA (86%), IAA (305%), ASA (170%), SA (87%), in *M. oleifera* plants under PEG-induced drought stress, in comparison to non-inoculated control plants under stress (**Figure 7**).

In contrast, endogenous ABA (-60%) and ACC (-77%) (an ethylene precursor) were significantly (*p ≤* 0.05) lowered in response to co-inoculation of TP and TR isolates in *M. oleifera* plants at 35 DAG, compared to non-inoculated control plants under stress (**Figure 7**).

### Effect of TP and TR endophytes on the stem anatomical features and water potential of *M. oleifera* under PEG-induced drought stress

Since water perturbances modify the anatomical features of plant parts such as the stem. For a better understanding of the influence driven by fungal endophytes (TP and TR) on the drought stress tolerance of *M. oleifera*, the cross-sectional investigation of the stem parts was performed by microscopic visualization. The observation of stem cross-section exhibited the conventional characteristics related to drought tolerance such as small cell gaps, water-filled cells, and tight and round cells under normal water conditions, as well as under drought stress conditions, upon co-inoculation by TP and TR. These typical features were lacking in the stem sections obtained from *M. oleifera* without endophytic association under drought stress, where they showed marginally contorted mesophyll cells, shorter epidermal as well as vascular bundle sheath cells, with narrower, phloem, metaxylem, cortical and pith area (**Figure 7**). Lactophenol cotton blue staining used to examine the root colonization potential of endophytic fungi, indicated the successful plant microbe-interaction in the root tissue of *M. oleifera* plants under observation (**Figure 7**).

Under drought stress, water potential was significantly (*p ≤* 0.05) reduced in the absence of individual as well as co-inoculation of TP and TR isolates in *M. oleifera* plants at 35 DAG, compared to non-inoculated control plants, with the greatest drop in water potential (-302%). While the lesser reduction in water potential was found in the water potential of *M. oleifera* plants with individual TP and TR isolates, in contrast, to control plants under stress. However, compared with non-inoculated, untreated control plants, the co-inoculation of TP and TR lead to the highest significant (*p ≤* 0.05) decline in reduction of water potential (-2%) in *M. oleifera* plants under drought stress (**Figure 7**).

### Effect of TP and TR endophytes on the antioxidant potential of *M. oleifera* under PEG-induced drought stress

**Figure 8:**
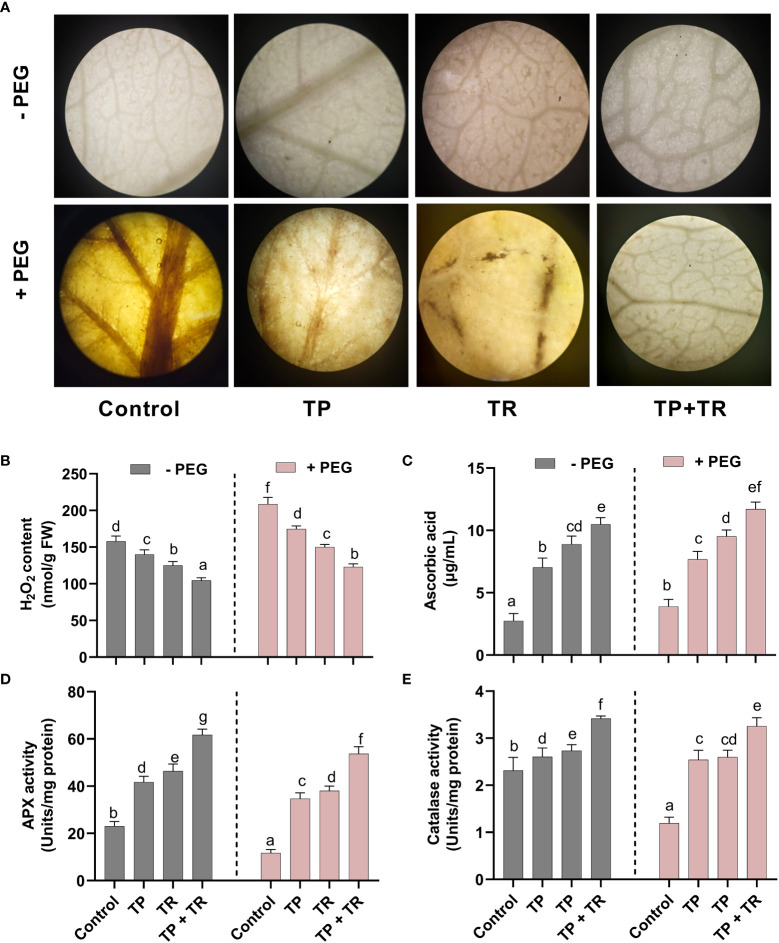
Effect of TP and TR inoculation on *M. oleifera* ROS production and antioxidant potential. **(A)** DAB staining using leaf segment from 35-d-old plants, **(B)** H_2_O_2_, **(C)** AsA, **(D)** Peroxidase activity and **(E)** Catalase activity. Quantitative data is representing the means ± *SE* values of at least three independent biological replications. Various letters have been presented to show the statistical differences at significance level of *p¾*0.05 using Duncan’s Multiple Range Test (DMRT).

Drought-induced cellular damage was evaluated in the form of reactive oxygen species (ROS) generation, where H_2_O_2_ production was investigated in *M. oleifera*. The H_2_O_2_ production was detected qualitatively, as the intensity of brown spots produced by using DAB (3,3-Diaminobenzidine) staining in *M. oleifera* leaf tissues. DAB intensity was stronger in the *M. oleifera* leaf tissues from plants grown under drought stress. This phenotype was reversed by application of TP and TR single as well as co-inoculation in comparison to non-inoculated control plants under drought stress (**Figure 8A**).

Likewise, quantitative analysis of the H_2_O_2_ production also revealed a significant (*p ≤* 0.05) increase in PEG-treated *M. oleifera* plants, up to 31% higher than in the control. While lower H_2_O_2_ accumulation (-45%) was detected in comparison to the control *M. oleifera* plants upon co-inoculation of TP and TR isolates under drought stress (**Figure 8B**).

Assessment for antioxidants (enzymatic and non-enzymatic), under drought stress in *M. oleifera* plants inoculated with individual and combination TP and TR isolates, also showed the differential response under various treatments. *M. oleifera* plants with combined inoculation of TP and TR isolates showed a significant (*p ≤* 0.05) increase in ASA (non-enzymatic antioxidant) level up to 201%, as compared to non-inoculated control under drought stress (**Figure 8C**).

Similarly, co-inoculation of TP and TR isolates also significantly (*p ≤* 0.05) induced the peroxidase enzyme activity (3.25%) as compared to non-inoculated control under drought stress (**Figure 8D**). Likewise, catalase activity was also increased (166%) in *M. oleifera* by co-inoculation of TP and TR as compared to non-inoculated control under drought stress (**Figure 8E**).

### Multivariate evaluation of endophytic fungal effects on the *M. oleifera* growth and stress tolerance

**Figure 9:**
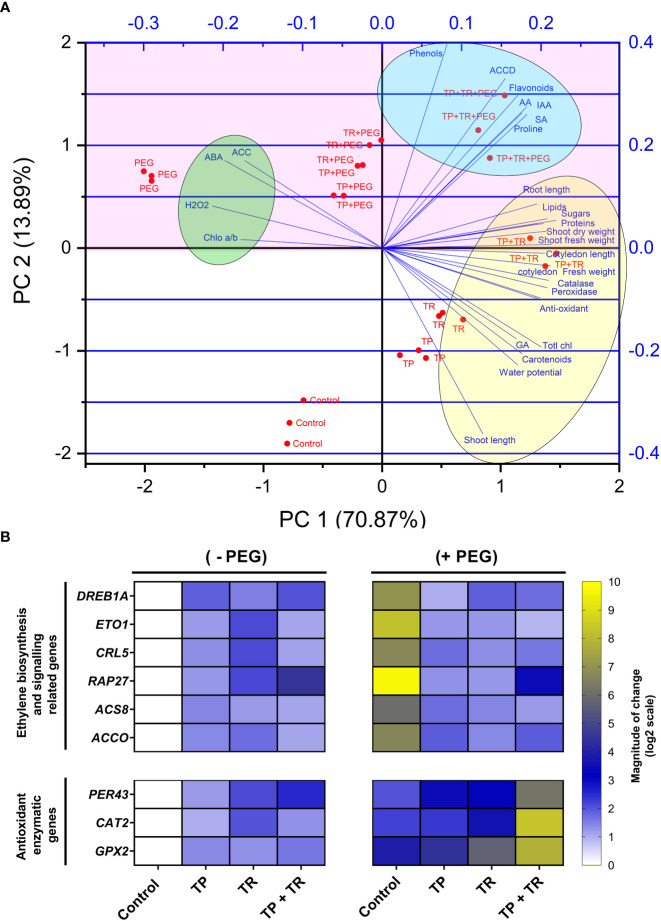
**(A)** Biplot of two dimensions of the principal component analysis (PCA). The scores show the contribution of TP and TR endophytic fungal isolates on reshuffling of *M. oleifera* plant traits, grown under normal and PEG-supplemented conditions, as indicated by the direction and magnitude of the respective vectors, and **(B)** Expression profiling of ethylene biosynthesis and antioxidant enzymatic genes by RT-qPCR. Quantitative data represent the means ± *SD* of three independent experiments and at least three technical replicates each.

The principal components analysis (PCA) deciphered the overall influence of endophytic fungi (TP and TR), on plant growth and stress tolerance behavior under PEG-induced drought stress. The PCA based on plant morphological, physiological, biochemical, hormonal, and antioxidant traits showed that the first two components PC1 (70.87%) and PC2 (13.89%) with the eigenvalue of 19.844 and 3.889, respectively. The PCA scores revealed a considerable contribution of endophytic fungi to the separation of physio-hormonal and biochemical features of plants under drought stress, as evidenced by the direction and amplitude of the corresponding vectors. The PCA biplot (**Figure 9A**) showed that all evaluated traits could be distinctively grouped into three major clusters. PC1 was positively and strongly correlated with phenotypic traits (cotyledon fresh weight, cotyledon length, shoot length, root length, shoot fresh weight, shoot dry weight), biochemical, metabolic, and hormonal traits (total chlorophyll, carotenoids, proteins, Phenols, sugars, flavonoids, lipids, proline, IAA, GA3, SA) of *M. oleifera*. Importantly, PC1 was also positively and strongly correlated with AsA (*r =* 0.18215), peroxidase (*r =* 0.21902), catalase (*r =* 0.21278), water potential (*r =* 0.17081), but negatively correlated with ABA (*r =* –0.197), H_2_O_2_, (*r =* –0.213), ACC (*r =* –0.172), and chlorophyll a/b ratio (*r =* –0.177).

### Gene expression analysis in *M. oleifera* co-inoculated with and TR under drought stress

The RT-qPCR study was performed to determine the degree of expression of chosen drought-induced marker genes related to ethylene biosynthesis and signaling (*DREB1A, ETO1 RAP27, ACS8, CRL5, ACCO)* and antioxidant enzymatic genes (*PER43, CATA2*, and *GPX2)* (**Supplementary Table 2**).

Current results exposed that *DREB1A, ETO1 RAP27, ACS8, CRL5, ACCO* showed considerably higher gene expression in moringa leaf tissues (>5-fold) in response to water deprivation in the absence of TP and TR association. While consortial inoculation of endophytes (TP and TR) under water deprivation reduced the expression of *DREB1A, ETO1 RAP27, ACS8, CRL5, ACCO* in leaf tissue to an optimal level compared to the control.

Current results also revealed that *PER43, CATA2*, and *GPX2* had considerably greater expression in moringa leaf tissues (>5-fold) in response to water deprivation by co-inoculation of TP and TR, in comparison to untreated control plants under drought stress (**Figure 9B**).

## Discussion

Environmental changes alter the endogenous phytohormonal levels, which modulate plant growth for better survival under stressful conditions. Unfortunately, not all plant species can quickly optimize their endogenous hormonal level in response to environmental perturbances. Researchers have developed a viable technique for sequestering biotic and abiotic stressors in plants by using Plant Growth Promoting Endophytic Fungus (PGPEF) and fungal communities. PGPEF has been shown to reduce stress in non-host plants as well, by boosting the threshold of tolerance to cope with hostile environments (Aziz et al., 2021a; Rauf et al., 2021; Ali et al., 2022a), however, the investigations are lacking at the molecular level for exposing and deciphering the role of endophytic fungal association with non-host plants to induce the tolerance against environmental stresses.

Endophytic fungi generate and exude a variety of secondary metabolites (alkaloids, phenols, terpenoids, and flavonoids), which increase the activity of antioxidant enzymes and activate a specific metabolic route that is considered to improve resistance against abiotic stresses (Mishra and Chan, 2016; Bilal et al., 2018; Rauf et al., 2022). Moreover, endophytes that produce growth-promoting hormonal contents have long been thought to be the ideal aspirants for plant growth promotion. IAA and GA_3_ are highly essential phytohormones in plants as it govern their developmental process and growth. While ABA, SA, and ethylene have been involved in abiotic and biotic stress alleviations through various cross-talks in plants (Sehar et al., 2022).

The existing study demonstrated the strong ability of ACC deaminase producing and growth-promoting endophytic fungal isolates, *Trichoderma gamsii* (TP), *Fusarium proliferatum* (TR), to exudate the IAA, GA, total phenols, proline, proteins, flavonoid, and lipids, representing its ability for inducing growth responses in non-host plants.

Remarkably, in the current study, it is revealed that IAA, GA, proline, total phenols, flavonoid, proteins, and lipids were considerably generated when TP and TR endophytes were subjected to PEG-mediated water deficit, making them ideal as water deficit ameliorating endophytes for plants. Importantly, both ACC deaminase enzymatic activity and gene expression were enhanced in *Trichoderma gammii* (TP) and *Fusarium proliferatum* (TR). There has been no earlier evidence that introduces ACC deaminase generating endophytic strain *Trichoderma gamsii* or *Fusarium proliferatum* as osmotic or water stress resistant microorganisms.

Only a few prior studies have shown that endophytes in plants may induce water deficit resistance, such as *Epichlo* promoted drought tolerance in *F. arundinacea* and *L. perenne* (Decunta et al., 2021), consortia of *P. chrysogenum, P. Phaeosphaeria*, *Alternaria* sp., *brevicompactum*, and *E. osmophilum* provoked drought tolerance response in *C. quitensis* (Hereme et al., 2020), co-inoculation of fungal isolates (SMCD 2206, 2210, and 2215) from *Ascomycota* induced resistance against water deficit in wheat (Hubbard et al., 2012). *Phoma* sp association improved seedling growth of *P. tabulaeformis* under water deficit, according to Zhou et al., 2021. Previously, the growth improvement and drought resistance of hybrid poplar upon supplementation with endophyte consortia has been reported (Khan et al., 2016). Langeroodi et al. (2020) reported the arbuscular mycorrhiza that protected the chicory (*C. intybus* L.) against water deficit and resulted in reduced photosynthetic efficiency. Different arbuscular mycorrhizal fungi have been reported to improve the growth and drought tolerance of *C. Migao* seedlings (Xiao et al., 2022). Halo et al. (2020) found that an endophytic fungus (*T. omanensis)* enhanced the physiological, biochemical, and anatomical characteristics of tomato plants under drought stress. Recently, Javed et al. (2022) reported a drought tolerance induction response of endophytic fungal consortia *M. majus* (WA), *M. guilliermondi* (TG), and *A. aculeatus* (TL3) supplemented to non-host plant Moringa under water deficit. However, none of these endophytes were investigated for ACC deaminase activity under water deficit. The current study is the first to investigate the newly isolated, water deficit-tolerant potential of ACC deaminase-producing endophytic strains *Trichoderma gammii* (TP) and *Fusarium proliferatum* (TR) isolates that demonstrated remarkable PEG-mediated drought resistance by enhancing sufficient biomass and growth-promoting metabolites.

Water deficit produces a significant drop in relative water content, a decrease in leaf water potential, turgor loss, and a reduction in cell expansion, resulting in a reduction in photosynthetic activity, distraction of various metabolic pathways, ROS burst (Boyer, 1982; Kohli et al., 2019) that destroys macromolecules (DNA, RNA, proteins, lipids, and carbohydrates), and eventually cause in irreparable destruction and cell death (Bali and Sidhu, 2019). Nonetheless, antioxidants such as superoxide dismutase (SOD), peroxidase (POD), and catalase (CAT) are vital in the elimination of ROS. During several abiotic stresses like metal and drought, ethylene harmonizes with ROS inventory (Chen et al., 2019). ERFs are involved in the regulation of ROS production and signal transduction. Furthermore, during abiotic stress, AP2/ERF transcription factor mediates ROS and ethylene crosstalk. ROS levels are increased by ERF1, which inhibits the expression of ROS scavenger genes during abiotic stress, causing ROS overaccumulation and a loss of resistance to stresses (Wang et al., 2014). The role of ethylene and ROS in many biological processes such as root and root hair formation, as well as various biotic and abiotic responses, has previously been documented (Steffens, 2014). However, much more research is needed to understand the interplay of ethylene with other signaling events triggered under abiotic stress facilitating the plant-fungal association.

There are several reports of drought-resistant 1-aminocyclopropane-1-carboxylate (ACC) deaminase-producing growth promoting rhizobacteria proficiently hydrolyzing ACC to – ammonia and ketobutyrate leading to reduction in ethylene production and growth promotion under environmental constraints and stressful conditions including drought (Heydarian et al., 2016; Tiwari et al., 2018; Duan et al., 2021). However, there are only a few reports have shown previously for ACC deaminase-producing fungal endophytes associated with the abiotic stress tolerance in plants. For example, the favorable impact of the ACC deaminase generating fungal endophyte *Trichoderma asperellum* (MAP1) in plant-microbe interaction for improving waterlogging stress tolerance in wheat has recently been examined by Rauf et al. (2021). *T. asperellum T203* produces ACC deaminase, which modulates endogenous ACC levels, promotes root elongation, and improves plant tolerance to abiotic stress (Viterbo et al., 2010).

Previously, several fungal species were known to have ACC deaminase ability to cleave the ET precursor ACC to a-ketobutyrate and ammonia due to the presence of the ACCD gene, but their role in inducing drought tolerance in plants is unknown. The presence of the ACC deaminase gene in the genomes of several species of the *Penicillium* and *Trichoderma* persuaded the control of ET synthesis by the fungus ACC deaminase, which might be thus associated with the plant resistance response to numerous forms of biotic and abiotic stimuli (Harman et al., 2004).

Endophytic fungi have been widely investigated in terms of their impact on the transcriptome, metabolome, and proteome of nonhost plants (Nogueira-Lopez et al., 2018; Ali et al., 2021; Aziz et al., 2021b; Ali et al., 2022b). Recently, Javed et al. (2022) explored the beneficial impact of endophytic fungus on water deficit mitigation in *M. oleifera.* However, the involvement of ACC deaminase-generating endophytic fungus associated with the non-host *M. oleifera* plant for water deficit adaptability has not been explored, particularly through bio-reduction of ET by endophytic microbe’s ACC deaminase activity.

According to the researchers, *M. oleifera*, a potential pharmacologically and nutritionally rich tree, demonstrated reduced overall growth and development when cultivated under 1.5 MPa water stress induced by polyethylene glycol (PEG-6000) (Boumenjel et al., 2021).

Given the significance of *M. oleifera* as a worldwide miracle tree, the present work was designed to improve water deficit resistance and growth performance of *M. oleifera* by efficiently colonizing the Morinaga amid water deficit with non-host ACC deaminase generating endophytic fungi.

Polyethylene glycol (PEG), a long-chain polymer with a wide range of molecular weights, causes water deficit in several plant species. It has been employed by researchers to measure agricultural drought resilience during seed germination because it affects seedling establishment and growth by reducing water potential, and the impact is more visible on shoots than on main roots. Several studies have demonstrated that *in vitro* screening with PEG is one of the most accurate methods for identifying drought-tolerant genotypes based on germination indices. Previously, PEG-induced water deficit has been shown to decrease the morphological, biochemical, physiological, and photosynthetic traits of plants including Moringa (Gururani et al., 2018; Javed et al., 2022; Mahpara et al., 2022).

Accordingly, current results showed that water deficit (8% PEG) significantly reduced the chlorophyll contents (chlorophyll a, chlorophyll b, and carotenoids) in plants which is attributed to reduced photosynthesis and decreased growth attributes (shoot and root length, fresh and dry weight) of *M. oleifera.* However, present results have also exposed that co-inoculation of ACC deaminase-producing endophytes (TP and TR) significantly reversed the drastic effect of water deficit and prominently promoted the growth attributes in *M. oleifera* plants under water deficit. TP and TR inoculation also promoted a decline in chlorophyll a/b ratio, as increased values of chlorophyll a/b ratio correspond to a decline in photosynthetic activity and initial stress symptoms in plants.

Our findings further suggested the induction of phenolics, flavonoids, and H_2_O_2_ that may be related to macromolecule breakdown inside plants during drought stress. Similarly, greater proline content in PEG-treated might be owing to faster protein breakdown and/or it is produced as a signal for osmoprotectant function. However, plants co-inoculated with ACC deaminase-producing endophytes (TP and TR) reversed this tendency, with further increased levels of proline (osmoprotectant), phenolics, and flavonoids, and showed improved osmoprotectant and antioxidant activities can be corroborated to enhanced stress tolerance in plants under stress. *M. oleifera* plants co-inoculated with TP and TR under PEG-stress, also showed higher production of protein, lipids, sugars, and lesser H_2_O_2_ content.

Previously, it is known that environmental stresses increase ROS production, especially H_2_O_2_, which must be controlled in a homeostatic pool (Ahmad et al., 2019; Mansoor et al., 2022); nonetheless, excessive ROS levels induce oxidative stress that causes protein denaturation, lipid peroxidation (by generating MDA; a product of lipid peroxidation in the destruction of biomembranes), nucleotide disruption, and may change plant physiology, eventually leading to plant death (Zaid and Wani, 2019; Raja et al., 2020). For minimizing the drastic effect of excessive ROS production, the plant system activates either enzymatic or non-enzymatic antioxidant processes. Endophyte inoculation also inhibited ROS formation in the plant cell by activating antioxidant enzymes, according to Redman et al. (2011).

The current study also revealed that co-inoculation of ACC deaminase-producing endophytes (TP and TR) improved the activity of ROS scavenging enzymes (POD and CAT) as well as non-enzymatic antioxidants (ascorbic acid) to overcome the effect of an excess of ROS (H_2_O_2_), while total antioxidant capacity was also increased, thereby overcoming oxidative stress in PEG-supplemented *M. oleifera* plants.

Under diverse abiotic stressors, ethylene biosynthesis is regulated by a feedback system that regulates ACO transcription (Argueso et al., 2007). The optimal level of ethylene aids in the reduction of reactive oxygen species (ROS) formation under different abiotic stimuli such as metal, drought, high salinity, low temperature, and so on (Iqbal et al., 2017). Furthermore, ethylene has a vital function in increasing ROS buildup, which acts as a signaling agent for inducing defensive machinery (Wi et al., 2010). The downregulation of superoxide dismutase (SOD) and peroxidase (POD) (ROS scavenger enzymes) expression by *ERF1* is the cause of the higher ROS level (Wang et al., 2014). However, the involvement of ethylene in GSH production in Arabidopsis thaliana under ozone stress has already been established (Yoshida et al., 2009). Thus, ethylene serves a dual purpose in decreasing optimum ROS generation in response to stress. Under water deficit, ethylene regulates stomatal closure *via* flavonol synthesis in guard cells *via* an EIN2-mediated mechanism by inhibiting ROS generation (Watkins et al., 2014; Zhang et al., 2016).

In both normal and stress conditions, ethylene guides plant growth and photosynthesis. Water deficit increased the levels of ethylene in several plant species, including faba bean, orange, french bean, and many more (Murti and Upreti, 2007). ROS generation and signaling in plants are principally controlled by ethylene-responsive transcription factors, i.e., the AP2/ERF gene family, which connects ethylene and ROS signaling under diverse abiotic stressors (Wang et al., 2014). In Arabidopsis with a constitutive promoter *35S:ERF1* demonstrated drought tolerance because of ERF protein interaction with the RD29B promoter’s DRE region (Cheng et al., 2013). For ethylene biosynthesis, OsETOL1 interacts with the type II ACS enzyme OsACS2. Rice eto1 mutations have an insensitive ETHYLENE OVERPRODUCER 1-LIKE (OsETOL1) protein with a higher appropriate ethylene level, which assists in plant survival under drought conditions, but rice overexpressing *OsETOL1* has lower ethylene, making it less drought-resistant (Du et al., 2014).

However, ethylene is also known to have a deleterious influence on plants when subjected to certain conditions. In Arabidopsis, for example, ethylene production rises under Cd stress due to the accumulation of *ACS2* and *ACS6* transcripts. *Arabidopsis acs2-1 acs6-1* double mutants exposed to Cd had a low amount of ethylene followed by a favorable reaction on leaf biomass (Schellingen et al., 2014).

Furthermore, the detrimental function of ethylene in alfalfa (Medicago sativa) exposed to mercury was investigated using 1-MCP (ethylene response receptor blocker) (Montero-Palmero et al., 2014). Similarly, Cu has been shown to stimulate the expression of *ACO1* and *ACO3* genes in *Nicotiana glutinosa* (Kim et al., 1998), which helps in the generation of ethylene (Keunen et al., 2016). The drought-induced metabolic pathways in *A. thaliana* have been linked to ABA-dependent and ABA-independent mechanisms driving drought-inducible gene expression (Guimarães-Dias et al., 2012), as well as a linkage between both signaling pathways (Kizis and Pagès, 2002).

Furthermore, improved ABA and ethylene signaling research have indicated that when both hormones are stressed, they function antagonistically among yield-impacting activities (Wilkinson et al., 2012). Although ethylene has been widely investigated in the process of plant senescence, its involvement in drought-induced senescence is less well understood.

Under drought circumstances, ethylene has been shown to produce leaf abscission and, as a result, reduced water loss (Aeong Oh et al., 1997). Under water stress, ethylene production was accompanied by a rise and then a drop in ACC, suggesting that water stress encouraged the *de novo* synthesis of ACC synthase, the rate-controlling enzyme along the ethylene biosynthesis pathway. Furthermore, ethylene and its metabolic pathway have a role in stimulating plant responses to flooding and water scarcity (Habben et al., 2014). It activates a signal transduction network, which results in the creation of many transcription factors that control gene activation/repression under stress, such as *ERF1* (Xu et al., 2007).

Several studies have shown that fungal endophytes can provide the plant with long-term resistance to biotic and abiotic stresses by balancing the various phytohormone-dependent pathways, the most important of which are salicylic acid (SA), jasmonates (JA), ethylene (ET), abscisic acid (ABA), auxin (indole-3-acetic acid: IAA), gibberellins (GA), and ethylene (ET), which modulate the levels of growth (Hermosa et al., 2013; Colebrook et al., 2014; Rauf et al., 2021).

Endophytic co-inoculation of ACC deaminase generating endophytes (TP and TR) not only created and supplied IAA, GA, and SA levels but also stimulated their production and accumulation in drought-stressed *M. oleifera* plants. Furthermore, inoculation of ACC deaminase generating endophytes (TP and TR) lowered ACC content, implying a reduction in ethylene production to an optimum level adequate for controlling the cell signaling initiation for induction of water deficit tolerance in drought-stressed *M. oleifera* plants.

Water stress caused quick and strong stomatal closure, which was fueled by abscisic acid (ABA) production, leading to photosynthesis suppression and negative effects on biomass production in *M. oleifera*, as stated previously (Brunetti et al., 2018). The phytohormone ABA regulates stomatal movements in plants, allowing them to preserve their leaf water potential and relative water content during drought stress (Coupel-Ledru et al., 2017).

Current research has consistently demonstrated that ABA overproduction in *M. oleifera* plants growing under drought stress may undergo extended stomatal closure, which inhibits photosynthesis, resulting in poor sugar production and producing less biomass. Endophytic inoculation of TP and TR isolates ably modulated the ABA production in *M. oleifera* plants under drought stress might be leading to optimally activated stomatal closure, leading to sufficient photosynthetic activity in plants under drought stress, as recently discussed by Javed et al. (2022).

The principal components analysis (PCA) is perfect for analyzing and exposing relationships between a large number of observable variables and a smaller number of elements. Inoculation of endophytes and plant characteristics under water deficit (Tabachnick and Fidell, 2007). The statistical assessment of the influence of endophytic fungal strains (TP and TR) on *M. oleifera* plants under water deficit and normal conditions was evaluated using the PCA approach, revealing differential responses of *M. oleifera* plants driven by ACC deaminase producing endophytic fungi under normal and water deficit conditions.

It is exposed from the PCA outcome that the first two major components offered sufficient information to support the main findings of the current study. Previous research has shown that chlorophyll a/b ratio, antioxidants, ROS, proline, ACC, ABA, phenolics, and flavonoids are good predictors of water deficit tolerance in plants.

This study also shows that rebalancing POX, CAT, and H_2_O_2_ levels are substantially linked with a variety of biochemical and physiological markers, including ACC levels. Furthermore, proline, photosynthetic pigments, peroxidase, catalase, ascorbate, phenols, flavonoids, carotenoids, lipids, sugars, proteins, and phytohormones (GA_3_, IAA, and SA) might be employed as further water deficit resistance markers.

The characteristics were shown in a biplot at the control and water deficit conditions. When compared to non-inoculated plants, *M. oleifera* associated with endophytes grown under control and water deficit circumstances projected diametrically opposite ends of the biplots.

More recently, the molecular analysis revealed that the expression levels of the genes associated with antioxidant, proline synthesis, ABA synthesis/signaling, and ethylene synthesis/signaling differed significantly between the wild-type and *ACC oxidase* mutants of petunia, indicating the role of ethylene in the transcriptional regulation of the genes associated with abiotic stress tolerance including drought stress (Naing et al., 2022). Consistently, the present research also showed the expression modulation of drought stress-responsive marker genes including ethylene biosynthesis and signaling-related genes such as *DREB1A, ETO1 RAP27, ACS8, CRL5, ACCO.* Moreover, orthologue genes for antioxidant enzymes such as *PER43, CATA2*, and *GPX2* were found to be predominantly induced by ACC deaminase-producing endophytic fungi (TP and TR) in *M. oleifera* under drought stress.

This modulation in ethylene biosynthesis and signaling genes as well as antioxidant enzymatic gene expression by ACC deaminase-producing endophytic fungi, to an optimal level, might be crucial for the *M. oleifera* plants under drought stress, hence is suggestive of a critical role in regulating the drought stress tolerance.

## Conclusion

In the present study that ACC deaminase-producing fungal endophytes (TP and TR) not only sufficiently produced endogenous growth-promoting regulators but also induced the basal level of growth-promoting and stress alleviating metabolites in *M. oleifera* plants under drought stress. The current study concludes with an emphasis on the potential of TP and TR endophytic fungi in the growth promotion of *M. oleifera* by adequate production of IAA, GA_3_, phenols, flavonoids, with increased chlorophyll content, resulting in greater biomass. Drought stress tolerance was also found to be induced due to the lowering of ethylene production to an optimal level, and accelerating antioxidant activities of enzymatic and nonenzymatic entities such as AsA, POD, and CAT in *M. oleifera* plants under drought stress. These findings also revealed the reshuffling of gene expression and modulation in the result of the fungal-plant association triggering the optimal ethylene biosynthesis and increased POX and CAT activity in *M. oleifera* plants under drought stress.

Finally, the current findings may be useful not only for the scientific communities of the pharmacological and pharmaceutical industries but also for farmers and forest scientists in the agroforestry industry to use this newly reported endophytic consortium as a bioengineer and bio-stimulant for the alleviation of drought stress in *M. oleifera* to promote sustainable agroforestry in drought-prone regions around the globe. Moreover, the current study also suggests that TP and TR endophytic fungi could be employed as biofertilizers for active crop production and stress reduction, particularly in arid regions with dry and xeric conditions.

## Data availability statement

The datasets presented in this study can be found in online repositories. The names of the repository/repositories and accession number(s) can be found below: https://www.ncbi.nlm.nih.gov/genbank/, OP419488 for TP isolate https://www.ncbi.nlm.nih.gov/genbank/, OP419489 for TR isolate.

## Author contributions

MR, SK, MA, and MH, conceived the idea and designed the experiments. BR, JJ, MR performed the main experiments. MR, SK, wrote the manuscript with inputs from all collaborators. MA, HG and MH, reviewed the manuscript. SAK, ZS, helped in biochemical studies and quantitative analysis. W-CK and I-JL reviewed the manuscript and provided the financial support. All authors contributed to the article and approved the submitted version.

## Funding

The authors declare that the current research was supported by the National Research Foundation of Korea (NRF) grant funded by the Korean government (MSIT) (No. 2022R1A2C1008993).

## Conflict of interest

The authors declare that the research was conducted in the absence of any commercial or financial relationships that could be construed as a potential conflict of interest.

## Publisher’s note

All claims expressed in this article are solely those of the authors and do not necessarily represent those of their affiliated organizations, or those of the publisher, the editors and the reviewers. Any product that may be evaluated in this article, or claim that may be made by its manufacturer, is not guaranteed or endorsed by the publisher.
