# Supplementary material for: ACC deaminase-producing endophytic fungal consortia promotes drought stress tolerance in M.oleifera by mitigating ethylene and H2O2
Source: Front Plant Sci. 2022 Dec 22;13:967672. doi: 10.3389/fpls.2022.967672 (PMC9814162; doi:10.3389/fpls.2022.967672)
Supplement: Supplementary file 1 [file Table_1.docx]

**Supplementary Table 1. Experimental setup with treatments for plant bioassay**

| S.No. | Treatment |
| --- | --- |
| 1. | Control + irrigation water |
| 2. | Plants treated with 8% PEG |
| 3. | Plants treated with TP |
| 4. | Plants treated with TP + 8 % PEG |
| 5. | Plants treated with TR |
| 6. | Plants treated with TR+ 8 % PEG |
| 7. | Plants treated with TP+TR |
| 8. | Plants treated with TP+TR+ 8 % PEG |
