# Supplementary material for: ACC deaminase-producing endophytic fungal consortia promotes drought stress tolerance in M.oleifera by mitigating ethylene and H2O2
Source: Front Plant Sci. 2022 Dec 22;13:967672. doi: 10.3389/fpls.2022.967672 (PMC9814162; doi:10.3389/fpls.2022.967672)
Supplement: Supplementary file 2 [file Table_2.docx]

**Supplementary Table 2. Selected Drought Induced Marker Orthologue Genes in *M. oleifera***

| **UniProt ID** | **Description** | **Orthologue** |
| --- | --- | --- |
| Q9M0L0 | *DREB1A (Dehydration-responsive element-binding protein 1A)* | *A. thaliana* |
| O65020 | *ETO1 (Ethylene-overproduction protein 1)* | *A. thaliana* |
| Q84Z02 | *CRL5 (AP2-like ethylene-responsive transcription factor)* | *O. sativa* |
| Q9SK03 | *RAP27 (AEthylene-responsive transcription factor)* | *A. thaliana* |
| Q9T065 | *ACS8 (1-aminocyclopropane-1-carboxylate synthase 8)* | *A. thaliana* |
| Q9MB94 | *ACCO (1-aminocyclopropane-1-carboxylate oxidase)* | *P. mume* |
| Q9SZH2 | *PER43 (Peroxidase 43)* | *A. thaliana* |
| P25819 | *CATA2 (Catalase 2)* | *A. thaliana* |
| O04922 | *GPX2 (glutathione peroxidase 2)* | *A. thaliana* |
